# Examining Knowledge Diffusion in the Circular Economy Domain: a Main Path Analysis

**DOI:** 10.1007/s43615-022-00189-3

**Published:** 2022-07-07

**Authors:** Abderahman Rejeb, Karim Rejeb, Suhaiza Zailani, Yasanur Kayikci, John G. Keogh

**Affiliations:** 1 Department of Logistics and Forwarding, Széchenyi István University, Győr, 9026 Hungary; 2 Faculty of Sciences of Bizerte, University of Carthage, Tunis, Tunisia; 3 Faculty of Business and Accountancy, Department of Operations Management and Information System, University Malaya, 50203 Kuala Lumpur, Malaysia; 4 Department of Engineering and Mathematics, Sheffield Hallam University, Sheffield, UK; 5 Science Policy Research Unit, University of Sussex Business School, Brighton, UK; 6 Henley Business School, University of Reading, Greenlands, RG9 3AU Henley-on-Thames UK

**Keywords:** Circular economy, Sustainability, Industry 4.0., Eco-innovation, Knowledge diffusion, Main path analysis

## Abstract

The circular economy (CE) field has recently attracted significant interest from academics and practitioners. CE represents a departure from the linear economy, which is characterised by unsustainable resource production and consumption. The growing number of publications necessitates a comprehensive analysis of this field. This is the first systematic examination of the knowledge base and knowledge diffusion pathways in the CE domain. We analyse a Web of Science dataset containing 5431 articles published between 1970 and 2020. To create a comprehensive review of the CE domain, we conducted a keyword co-occurrence network analysis. We examined four distinct types of main paths using the main path analysis (MPA) technique: forward, backward, global, and key-route. According to the analyses, CE research focuses on six primary research themes: CE and sustainability, bioeconomy, CE practices, lifecycle assessment and industrial symbiosis, construction activities, and waste management. In addition, the MPA demonstrates that the CE literature has recently focused on Industry 4.0 technologies and their contribution to CE. This is the first attempt to depict the genealogy of CE research so that scholars can comprehend the domain’s evolutionary structure, identify hot topics, and capture the history, development status, and potential future directions of CE research.

## Introduction

Today’s industries face significant economic, environmental, and social challenges due to rapidly changing conditions. Specifically, rising resource scarcity, soil contamination, climate change, and environmental risks compel businesses to adopt a circular economy (CE) [115]. Historically, the linear economy has produced excessive waste throughout the supply chain, from raw materials to storage, processing, transportation, and packaging, which all end up in landfills [62, 93]. According to Blomsma et al. [19], the linear economy is frequently characterised by structural waste, which consists of components, products, or materials that reach the end of their useful life prematurely or have their value creation capacity underutilised. The constraint of the linear economy approach is its exclusive focus on the “take-make-use-dispose” cycle, which may be insufficient for resource efficiency [131]. The make-use-dispose model strains natural ecosystems by consuming finite resources excessively and generates large amounts of waste that must be avoided [106]. To address these issues, the CE is designed as a regenerative system in which the value and utility of materials are maximised throughout their lifecycle, and resource input, waste, emissions, and energy leakage are minimised through closed energy and material loops [80, 130].

Adopting the CE can result in many tangible and intangible benefits, including new opportunities for collaboration, market access, product and service innovation, waste minimisation, and long-term value retention. Moreover, the CE improves environmental performance and reduces the risks of resource scarcity [115]. CE implementation entails adding value to materials and products and renewing them until they reach the end of their useful life to maximise their life cycle [86]. Further, the CE aims to develop a closed-loop system that minimises resource inputs, emissions, and waste. To that end, the CE aims to eliminate waste, maximise resource efficiency, and achieve a harmonious balance between society, the economy, and the environment by closing the life cycle of products. Nonetheless, achieving these goals will be difficult, as the transition to the CE will require financing, economic enablers, technical capabilities, and significant (if not radical) changes in consumer behaviour, business models, institutions, and governance [109]. While some businesses are implementing circularity strategies and increasing their offerings of circular products and services, widespread adoption of CE is still lagging [5].

Since the introduction of the concept of CE, researchers working on eco-industrial initiatives and environmentally responsive economies have paid increased attention to it. Due to the rapid increase in research activity and interest in the CE domain, this area calls for systematic investigation and analysis. Schöggl et al. [147], Khitous et al. [82], and Goyal et al. [61] have all reviewed this area and used bibliometric analysis, content analysis, and longitudinal bibliographic network analysis to analyse various research themes and trends. While they contain valuable information about the CE, to our knowledge, no paper has examined the knowledge diffusion trajectories of the CE domain using the main path analysis (MPA) method. Hummon and Dereian [67] initially proposed the MPA to capture the most critical knowledge route at each conjecture of knowledge diffusion for a scientific field. By visually representing and tracking the evolution of research and specific academic hotspots, the MPA aims to strengthen the citation links between the literature data and gain a more detailed understanding of the CE domain. The advantage of MPA is that it can simplify a complex citation network by retrieving significant paths within it [28]. This strategy is widely used in various research fields, including information technology outsourcing, vehicle research, and blockchain [97, 174, 178].

The studies referenced above shed significant light on the CE domain. However, to the best of our knowledge, no systematic research has been conducted to examine the CE domain’s progress comprehensively through the MPA. Thus, the evolution of knowledge in the CE domain through the MPA method is timely. As such, this study seeks to address the following research questions:

1. What are the characteristics of publications in the CE domain?
2. What are the trending topics in this domain, and how have they evolved in the past years?
3. How does the knowledge diffuse, and what is the knowledge development process in this domain?

The primary objective of this study is to conduct a keyword co-occurrence analysis, a content analysis of the most recent influential works, and the MPA on the entire domain of CE. Analysing the keyword co-occurrence network is a valuable and helpful approach to exploring the progress of research topics in the CE knowledge domain [182]. Moreover, this network can uncover the collective interconnection of terms and the research hotspots [39]. The content analysis of the most recent influential works is performed to understand the current trend and future research directions.

The use of MPA assists in identifying knowledge diffusion pathways and identifying the significant factors influencing the historical development of the CE. Additionally, the knowledge transmission paths and map can be presented intuitively by using four distinct types of main paths. These include the local (forward and backward), global, and key-route main paths. These quantitative approaches contribute to objectivity, minimise analysis bias, and enable scholars to compile and investigate a large sample of selected papers more effectively. With the assistance of these methods, it is possible to enlighten and improve researchers’ understanding of the CE field’s historical development, ascertain the current state of research, and hypothesise on future trends.

This study adds to the existing body of knowledge in the following ways: This is the first known attempt to use the MPA to comprehensively reveal the knowledge diffusion paths of the CE domain. The MPA is a novel technique that was overlooked by previous researchers of the CE domain. Second, this article presents a framework for analysing the pathways of knowledge diffusion in this domain from various vantage points. In recent years, according to the MPA, the trajectories of knowledge diffusion have become clear, allowing them to be captured and thoroughly described in order to provide useful information for future research. In contrast to previous literature reviews on this domain, this study employs the MPA to analyse thousands of documents, enabling researchers to gain an objective and comprehensive understanding of the knowledge base and historical evolution of the CE domain.

The remainder of the article is structured as follows: In the “Data and Methodology” section, the data source and methods are introduced briefly. The “Content Analysis Based on Keyword Co-occurrence Clustering and Recent Works” section provides a content analysis based on keyword clustering and the most influential works published in 2020 and 2021. The “Findings of MPA” section is followed by the “Discussion” section. The final section concludes the paper.

## Data and Methodology

### Data

**Fig. 1:**
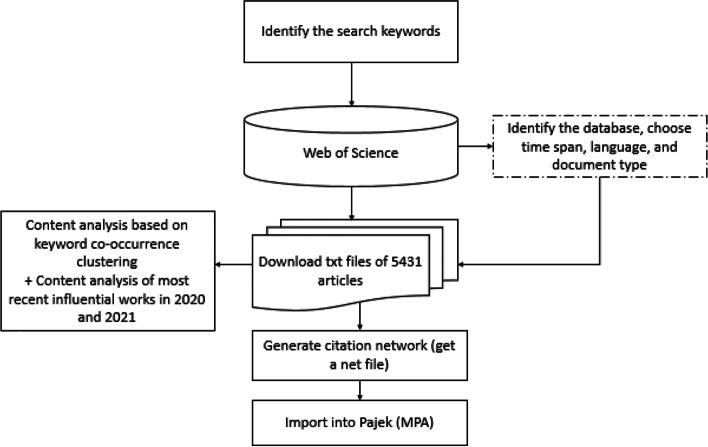
The review processes

Data quality is critical for producing meaningful results. Due to its comprehensiveness and high quality, it is argued that the Web of Science (WoS) is the most frequently used database for bibliometric studies [46]. According to Movahed and Sarmah [116], WoS provides intelligent tools for analysing the data in various formats, simplifying the process of removing irrelevant data and optimising the results. Apart from its widespread use in bibliometric studies, WoS is an effective tool for conducting the MPA in various research fields (e.g. [171, 178]. As a result, this paper uses WoS to identify all potentially relevant literature. The process for conducting this review is detailed in Fig. 1.

The title, abstract, and keyword fields are populated with the search term “circular economy” in reference to a seminal review on the circular economy by Kalmykova et al. [73]. This article utilises four databases in WoS, including Science Citation Index Expanded (SCI-EXPANDED) and the Social Sciences Index (SSCI). The article contains all journal articles (including reviews) published between 1970 and 2020, resulting in 5660 documents. After excluding 229 non-English language documents, 5431 articles were compiled and downloaded from the WoS database in response to the search query.

### Methodology

#### Content Analysis Based on Keyword Co-occurrence Clustering and Most Recent Papers

To gain a deep understanding of CE research, we generated a keyword co-occurrence network. Like co-citation networks, a keyword co-occurrence network reveals the respective relationships between co-occurring keywords [135, 137]. According to Su and Lee [156], a keyword co-occurrence network analysis helps researchers identify research topics and detect the hotspots or research frontiers in a given scientific field. In the network, a pair of keywords have a closer relationship if they co-occur in the same articles more frequently [135, 137]. By conducting this analysis, we aim to analyse the core content from the used keywords and portray the present structure of CE research. Because of its high flexibility and compatibility with the BibExcel tool, the computer program used to construct the network is VOSviewer [38]. In this network, the node’s size is proportional to the number of occurrences of each keyword, and the thickness of the edges reflects the number of times each pair of keywords appears together in articles. We analyse the content and critical topics discussed in CE research by clustering keywords. Further influential studies published in recent 2 years, from 2020 to 2021, are analysed separately to identify the current and potential research directions.

#### Main Path Analysis

Citations are critical resources for examining the spread of knowledge and assessing a scholar’s contribution to science. Thus, a citation index is an effective and efficient method for conducting citation analyses [49]. From a temporal perspective, a citation network is a time sequence graph that depicts the historical evolution and knowledge transmission of a research field. A citation network analysis provides insights into a particular research domain’s historical development and evolution or even across multiple research domains. Additionally, it provides an intuitive representation of the role of the literature and researchers and the types of theories and methods that are prevalent in a particular research field.

**Fig. 2:**
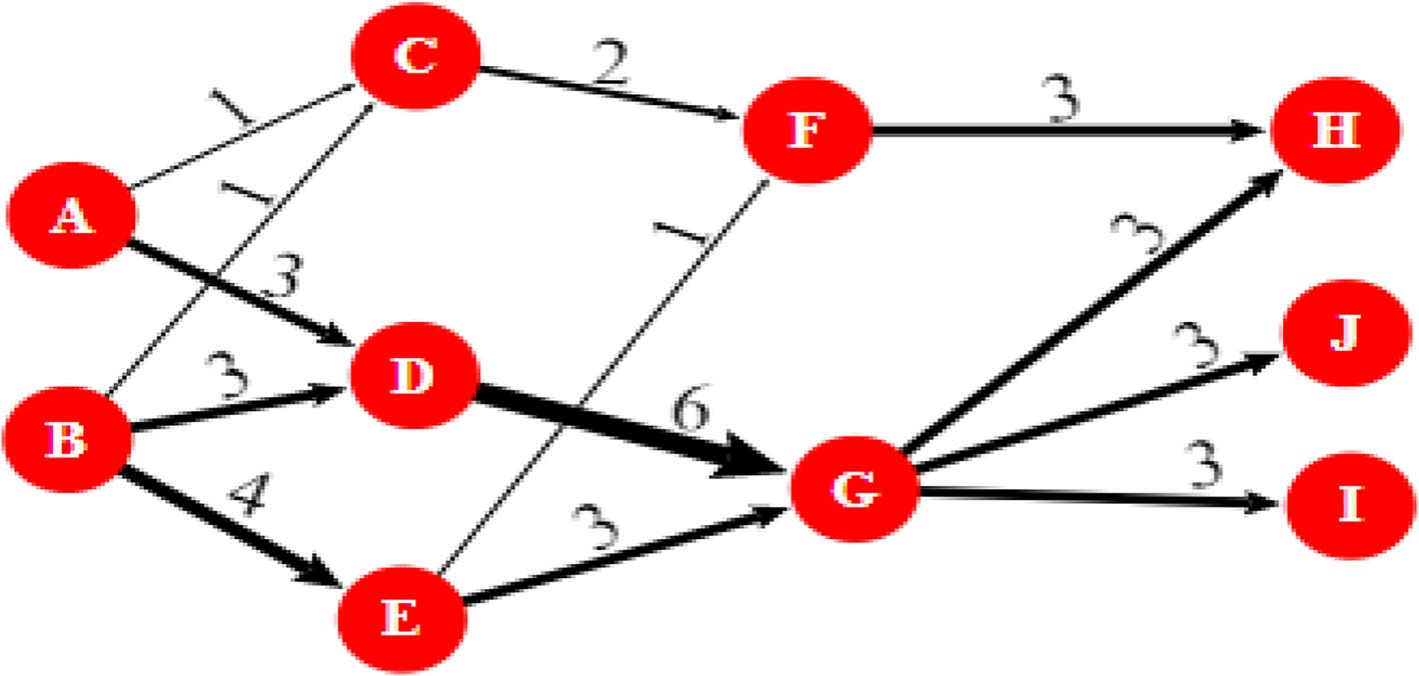
A typical citation network with SPC values

We used the MPA to analyse the citation network depicting the evolution of the CE field in this study. The MPA was initially introduced by Hummon and Dereian [67] as a technique for determining the knowledge flow between articles based on their direct citation connections. The MPA is also used in a variety of other fields due to its effectiveness, including environmental innovation [13], computer science [45], and transportation [174]. Following its introduction, Batagelj [14] developed a new algorithm called search path count (SPC) to facilitate the analysis of large citation networks. Liu and Lu [100] proposed novel methods for identifying different main paths. The current main paths involve local (forward and backward) main path, global main path, and key-route main path. This study illustrates how the citation network is weighted and how the main path is determined using a typical citation network, as shown in Fig. 2.

Each node constitutes an article in the citation network, and the edge from cited article to citing article indicates the route of knowledge flow. In Fig. 2, there are three types of nodes: source nodes (e.g. A and B), intermediate nodes (e.g. C, D, E, F, and G), and sink nodes (e.g. H, I, and J). The SPC value of each arc represents the total number of times the arc is traversed. For example, the SPC value of arc A-C is 1 since only 1 path passes through this link, that is, A-C-F–H. The SPC value of arc B-E is 4 since four paths pass through the B-E arc, namely, B-E–F-H, B-E–G-H, B-E–G-I, and B-E–G-J. The highest the value of the SPC is, the more critical the arc.

After building and weighting the citation network, different search algorithms are used to obtain different main paths. For instance, the forward local main path searches from sources to sinks. At each node apart from sinks, the arc with the highest SPC value among all arcs emanating from that node is chosen. For example, at node E, two arcs emanate from it, E–F and E–G. Arc E–G is chosen because its SPC is higher than arc E–F. Subsequently, node G is chosen as the starting node for the next search. This step is repeated until any sinks are reached. The forward local main path can be obtained, which is the combination of B-E–G-H, B-E–G-I, and B-E–G-J.

As opposed to the forward local main path, the backward local main path aims to search from sink nodes to source nodes, thus tracking back to the earliest articles and identifying the roots of the latest articles. Overall, 9 arcs connect to the sink nodes H, I, and J. Arcs with the largest SPC, 3, are chosen at the beginning. The search will terminate until any source nodes are reached. Therefore, the combination of paths A-C-F–H, A-D-G-H, A-D-G-I, A-D-G-J, B-C-F–H, B-D-G-H, B-D-G-I-B-D-G-J, B-E–F-H, B-E–G-H, B-E–G-I, B-E–G-J, and A-D-G and B-D-G are denoted as backward local main paths. In this illustrative example, A-D-G-H, A-D-G-I, A-D-G-J, B-D-G-H, B-D-G-I, and B-D-G-J are the global main path generated because their accumulated SPC values on these arcs are the highest among all paths from source nodes to sink nodes.

Liu and Lu [100] propose the key-route main path to improve the MPA and increase its robustness. The process of obtaining this path is as follows. First, the arcs with the highest SPC value in the network are identified as the target links, of which one can select more than one arc at one time. Second, searches from the end node of a target link are conducted to identify the most critical path forward. Then searches from the starting node of the same link are conducted to identify the most critical path backward. During this step, a local or global method can be used. Third, the forward and backward search outcomes are combined into one path to extract the key-route main path. Lastly, the second step is reiterated to work on the remaining target links until all of them are dealt with. For instance, in Fig. 2, the target link with the highest SPC, D-G, is selected as its SPC is the largest, 6. Then, the key-route main paths obtained are A-D-G-H, A-D-G-I, A-D-G-J, B-G-D-H, B-D-G-I, and B-D-G-J following the previous process. Multiple key-route main paths can be extracted. For instance, in the key-route 2 main path (the key-route main path based on the second-largest SPC), the second step is reiterated, and the B-E link is found to have the largest SPC value of 4 in the remaining links, and paths B-E–G-H, B-E–G-I, and B-E–G-J are extracted.

The advantage of the key-route main path method is its ability to generate several paths from which knowledge diffusion routes can be identified comprehensively. Additionally, this method consists of almost all significant links and makes the findings complete. While the local main paths help track the most important citation connection at every possible juncture, they may not yield the path with the highest overall impact. As a result, the global main path addresses the shortcoming of the local main path by finding the path with the highest accumulated traversal counts. However, these two methods may omit the arcs with the highest traversal count. As a potential solution, the key-route main path analysis begins by finding the link(s) with the highest SCP. This study applies the four different main paths to understand the knowledge diffusion routes in the CE field.

## Content Analysis Based on Keyword Co-occurrence Clustering and Recent Works

**Fig. 3:**
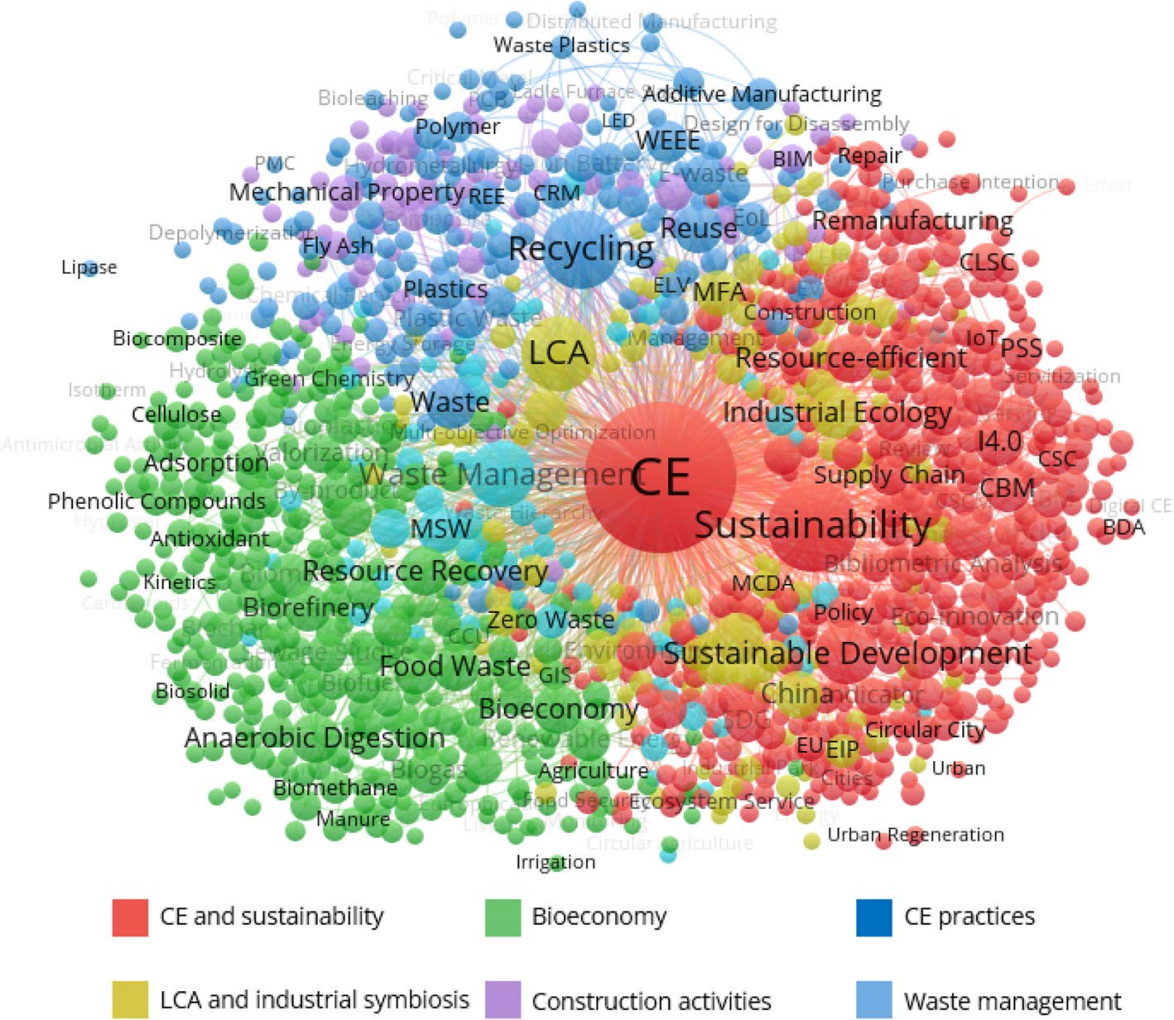
Keyword co-occurrence network

We carried out a keyword co-occurrence network analysis to identify the different research clusters in CE research. This aids us in uncovering the critical research foci that contributed to the CE field. We began by retrieving, reviewing, and refining the author-supplied keywords as the unit of analysis. The full-length keywords were refined and abbreviated (e.g. circular economy and CE). We loaded the data into the VOSviewer computer program to construct the network. Then, we used the density-based spatial clustering applying the full counting method, which calculates the total number of occurrences of a keyword in documents [87]. We fix the threshold of 5 keyword occurrences, resulting in a network with six clusters (see Fig. 3). In the network, each node corresponds to a keyword. The node’s colour denotes the cluster to which the node belongs. The size is proportional to the frequency of the keyword. Finally, the distance between nodes indicates the density. That is, the higher the density, the closer the nodes. Table 1 lists the top 10 most frequent keywords in each cluster.

**Table 1:** Top 10 most frequent keywords in each cluster

Cluster 1	Cluster 2	Cluster 3	Cluster 4	Cluster 5	Cluster 6
CE	Bioeconomy	Recycling	LCA	CDW	Waste management
Sustainability	Food waste	Waste	Industrial ecology	Mechanical property	MSW
Sustainable development	Resource recovery	Reuse	Industrial symbiosis	Sustainable construction	Waste-to-energy
Resource-efficient	Biorefinery	Plastics	China	Concrete	Zero waste
Business model	Anaerobic digestion	WEEE	MFA	Durability	Developing country
SDG	Biomass	Recovery	Environmental impact	Cement	Energy efficiency
I4.0	Wastewater	EoL	Building	Characterisation	Solid waste
CBM	Biogas	E-waste	Closed loop	Building material	Municipal waste
Environment	Renewable energy	Plastic waste	Optimisation	Construction material	Landfill
Remanufacturing	Waste valorisation	Packaging	Carbon footprint	Geopolymers	Machine learning

Figure 3 shows that the most important cluster is the red one. It is a generic cluster and highlights the potential of the CE for achieving sustainability and fostering sustainable development goals (SDGs). As a holistic approach, the CE aims to promote sustainable development at the local and global levels [52, 147, 157]. Keywords such as sustainability, sustainable development, resource-efficient, business model, SDG, circular business models (CBM), and remanufacture have occurred frequently within this research cluster. According to Geissdoerfer et al. [52], the CE enables the development of a closed-loop system that reduces all resource inputs, waste, and emission leakages. The CE is mainly stimulated by the fact that resources could be efficiently used and emissions and waste reduced with the embrace of circular business models rather than linear economy systems. Kumar and Saravanan [91] state that the CE focuses on achieving sustainability with the support of waste management and the implementation of zero waste. However, the transition from the linear economy to the CE requires a systematic change to current industrial systems considering all processes affected. In this regard, the espousal of Industry 4.0 (I4.0) facilitates CE implementation by enabling sustainable business models considering ethical dimensions of corporate social responsibility [35, 44, 48, 151]. Adopting Industry 4.0 technologies in the CE can strengthen the competitive advantage [173] because firms would be able to achieve sustainable and ethical manufacturing operations [61]. de Sousa Jabbour et al. [103] emphasise that sustainable development can only be realised by endorsing cleaner production practices. Industry 4.0 can unlock circular business models and sustainable manufacturing, reduce costs, support product customisation, and optimise operational efficiencies [92]. Industry 4.0 technologies can also be used to resolve remanufacturing issues by transforming production and consumption behaviour [5], predicting returns of products for remanufacturing activities [10], and improving product designs and characteristics [146]. Overall, researchers in this cluster have investigated the contributions of the CE to sustainable development and the role of Industry 4.0 in supporting circularity strategies, circular business models, and remanufacturing.

Bioeconomy has been identified as one of the important research clusters in the CE domain. Conceptually, the bioeconomy represents a complex mechanism that replaces the economy based on fossil, non-renewable natural resources [64, 94, 163]. As a new concept, the bioeconomy supports the sustainable production and transformation of renewable biomass into a spectrum of bio-based energy, chemicals, and products [36]. The application of bioeconomy first originated within the life sciences and biotechnology and subsequently expanded to cover other related ideas, including biorefineries [95, 127, 163, 168]. This research theme’s main terms and keywords are food waste, resource recovery, biorefinery, anaerobic digestion, biomass, waste, biogas, and renewable energy. Sustainable food waste management can facilitate the implementation of a circular bioeconomy since excreted and digested food products end up in energy recovery, waste recycling, or landfill disposal [111]. As a result, the overlap of CE and bioeconomy can enhance resource recovery and eco-efficiency, reduce greenhouse gas emissions, minimise fossil consumption, and valorise waste streams. Takala et al. [159] note that the frontiers of bioeconomy are broadened toward the reliance on renewable energy resources such as solar, wind, and sometimes geothermic energy. According to the authors, this makes the vision of the bioeconomy a more inclusive green economy since it relies on any sort of renewable resources and removes the requirement for the bio-prefix. However, the increasing need for biomass as part of the bioeconomy implementation is expected to result in a biomass shortage [23].

The third cluster emphasises the importance of several practices to achieve the CE. For example, CE policies have highlighted recycling and reuse as the starting point and focus. With the finite natural resources and the limited land for dumping, recycling has become a vital step in promoting the CE to transform wastes into novel resources and bridge the production and consumption loops [176]. Based on recycling and reuse processes, the CE has the potential to optimise sustainable economic growth, create new jobs for the economy, and encourage entrepreneurs and businesses to invest in sustainable activities [161]. The CE approach involves material reuse and recycling and the minimisation of consumption and waste [129]. The high frequency of the keywords “Plastics”, “WEEE” (waste electrical and electronic equipment), “E-waste” (electronic waste), and “plastic waste” suggests that CE research has widely focused on the recycling and reuse of plastic materials. In general, plastics are considered non-biodegradable and highly durable materials derived from petroleum products [59] 59, with a lifetime of up to thousands of years. Plastics’ durability and versatile nature make them essential, leading to their high demand and consumption worldwide. The heavy reliance on plastic materials exacerbates environmental pollution and affects various ecosystem elements [42, 113, 152]. As a result, the involvement of all CE stakeholders operating in the plastics value chain is necessary to close the loop, facilitate circularity, and reduce plastic waste [126]. The realisation of the CE of plastics requires the redesign of plastic processes and products to make them entirely recyclable and reusable. The plastics industry also needs to redesign packaging and implement novel models for exploiting packaging and maximising the recycling rate. These can be achieved, for instance, by developing efficient after-use plastic products, diminishing plastic leakages into the natural environment, and separating plastic materials from fossil raw materials.

The next research theme revolves around lifecycle assessment (LCA) and industrial symbiosis. The CE implies a change in the vision of production systems that overcome the existing negative economic impacts by creating new enterprises and jobs, thus driving the transformation of traditional linear economic systems and providing environmental and social benefits. This increasingly responsible and accountable approach can be incorporated into businesses’ decision-making processes through the LCA method [32, 72, 102]. LCA represents a technique for analysing the environmental impacts of each step of a product or a service across the whole value chain, from the extraction of raw materials through processing, manufacturing, distribution, usage, repair and maintenance, and recycling of disposal [21, 31, 144]. The LCA approach considers various indicators, including economic, social, and environmental variables, to provide a reliable analysis of the production system with certain effect categories. LCA intends to enhance recycling activities and integrate all the stages of the product lifecycle, comprising production, consumption, waste management, and the introduction of secondary raw materials, such as food waste, plastics, bio-based products, and biomass [30, 112, 123]. Researchers in this cluster also focused on the concepts of industrial ecology and industrial symbiosis. Like natural ecosystems, an industrial ecology system strives to increase the economic utilisation of products and wasted materials at the end of their lives (EoL) as inputs to other activities and industries [71]. Industrial activities can function as metabolism in industrial ecology, allowing various actors to be optimally integrated through their resources and wastes [130]. As a result, this can assist in the shift from the linear economy to the CE.

The cooperation among entities and organisations to share resources and increase sustainability has led to the development of industrial symbiosis. In this respect, Neves et al. [120] argue that industrial symbiosis is a key enabler for CE implementation since it balances economic growth and environmental protection. Kerdlap et al. [81] point out that industrial symbiosis supports interfirm symbiotic processes to motivate the development of mixed urban-industrial environments and eco-industrial parks that offer both economic and environmental benefits. Another approach used in industrial ecology is material flow analysis (MFA), which systematically assesses the flow of materials throughout the system and delivers a full scope of the material flow and inventory [167]. Finally, the high occurrence of the keyword “China” reflects the significant interest of researchers in applying LCA methodologies and exploring industrial ecology and symbiosis in the context of China.

The fifth cluster in purple is centred on the theme of construction within the CE domain. Highly frequent keywords include “CDW” (construction and demolition wastes), “Mechanical Property”, “Sustainable Construction”, and “Concrete”. The construction industry represents one of the biggest consumers of energy and materials [65]. According to the [41], construction activities account for 40 per cent of all GHG emissions, 50 per cent of all energy use, 30 per cent of all water use, and 50 per cent of all materials extracted. Because of the high CDW, optimised and integrated management of this waste is urgent to reduce emissions, resource depletion, and climate change by adopting the CE approach [57]. The shift toward more sustainable and circular construction activities, in which materials flows can be reintroduced as secondary resources, is a possible alternative for the construction sector. Novel business models altering the end-of-life concept by reusing, reducing, recovering, and recycling building materials in production and consumption processes are promising [84]. Moreover, recycling construction waste can help to boost the market for secondary construction feedstock [33]. The adoption of CE practices in construction activities can also foster environmental sustainability, particularly when CDW and asphalt shingle are recycled into new concrete or used to produce cement [6, 153]. As a sector with vast potential to support the CE transition, the construction industry introduced geopolymers, representing green and alternative cement materials with reduced environmental impact and superior performance [10, 99]. Overall, the focus of this cluster is on the necessity to establish a circular construction industry. This requires the continuous development of systems and tools for material reuse, recycling, and recovery and the formulation of schemes incentivising the stakeholders to invest in circular and closed-loop construction activities [57, 65].

The last cluster in aqua discusses the theme of waste management in the CE domain. The high frequency of “Waste Management”, “MSW” (municipal solid waste), “Waste-to-Energy”, and “Zero Waste” indicate the heightened importance of improving waste management by maximising the efficiency across the value chain in terms of production, consumption, and resources performance [7]. As a key toward sustainability, the management and valorisation of municipal solid waste constitute a critical matter for decision-makers due to the serious effects that faulty waste management systems can cause to society and the environment. Indeed, unregulated and unsuitable municipal solid waste treatment can damage soil, impair water quality, and accelerate global warming by increasing GHG emissions [1]. As a result, concerns over the diversion of municipal solid waste from landfilling to recycling attract researchers’ attention to alleviate the issues mentioned above and turn wastes into high-value-added resources. This can be achieved by implementing waste-to-energy solutions to facilitate waste disposal and energy recovery. Examples of waste-to-energy treatment include pyrolysis, anaerobic digestion, gasification, landfill gas recovery, and incineration [15]. However, the release of pollutants associated with the combustion of wastes remains the principal environmental downside of waste-to-energy activities [125]. Finally, the keyword “Machine Learning” reveals the benefits of this technology in predicting the generation and transfer of MSW, thus reducing the environmental impact of waste [104, 105].

To complement the keyword co-occurrence network analysis, a content analysis of the recent most influential publications of 2020 and 2021 is carried out to capture recent research trends in the CE domain. The selection of recent publications enables to uncover additional insights into the ongoing research trend, which might not be gained from the keyword clustering-based content analysis. According to Table 2, scholars started paying significant attention to the interplay between Industry 4.0 and the CE (Bag, Gupta, et al*.*, 2021; [58, 140, 173]. At a detailed level, researchers also tended to focus on the potential of specific Industry 4.0 technologies for enabling the CE transition, including blockchain technology [40, 117] and big data analytics and AI [9, 11]. Moreover, the impact of the COVID-19 pandemic on CE implementation is another research theme that has emerged as an actual trend driven by the need for sustainable production and consumption in the COVID-19 era [117]. Biorefineries, plastics, food waste, and e-waste management have also remained debatable in the recent CE literature.

**Table 2:** Content analysis of most recent influential works in 2020 and 2021

Author(s)	Year	Scope	Objective(s)	Main finding(s)	Knowledge gap(s) for future research
Ghobakhloo [58]	2020	Industry 4.0	To systematically identify the sustainability functions of Industry 4.0 and model the contextual relationships among them	The presence of sophisticated precedence connections among different sustainability functions of Industry 4.0 Economic sustainability functions of Industry 4.0, including business model innovation and production efficiency, represent the more immediate result of Industry 4.0, achieving energy sustainability, reduced emissions, and improved social welfare	The negative impacts of Industry 4.0 on sustainability and CE implementation The potential inequality effects of Industry 4.0 The impact of digital manufacturing innovation on employee performance and well-being in the CE context
Ubando et al. [163]	2020	Biorefineries	To review different biorefinery models utilised for different biomass feedstocks, including algae, lignocelluloses, and several waste-types	The social-economic dimension of the industrial sector has a significant impact on the adoption of biorefineries in the circular bioeconomy Biomass wastes contribute to the implementation of biorefinery in the CE	The potential of biorefinery systems for the transition toward circular bioeconomy Exploration of logistics issues during the integration of various feedstock sources, processes, and distribution in the context of circular bioeconomy
Vollmer et al. [169]	2020	Plastic waste	To summarise the different chemical recycling routes and evaluate through lifecycle analysis a list of processes of companies involved in chemical recycling	A combination of various technologies can resolve the plastic waste issue	Additional focus on improving the collection and sorting infrastructure of more pollutant and mixed waste streams
Yadav et al. [173]	2020	Industry 4.0	To propose a framework to address sustainable supply chain management (SSCM) issues through Industry 4.0 and the CE	Identification of 28 SSCM challenges and 22 solutions measures Managerial and organisational challenges and economic issues represent the most critical barriers to SSCM adoption	Investigation of Industry 4.0 and the CE-based solutions to SSCM issues in developed countries
Kristensen and Mosgaard [88]	2020	CE and sustainability	To review indicators of a CE at the micro-level	Most indicators focused on recycling, remanufacturing, or end-life management, while fewer indicators reflect on lifetime extension, disassembly, reuse, waste management, and reuse There are no universally recognised means of assessing CE at the micro-level or within the various CE concepts of remanufacturing and recycling	Explore the indicators of the CE from the meso and macro perspective
Rosa et al. [140]	2020	Industry 4.0	To assess the relationship between CE and Industry 4.0	An innovative framework highlighting the links between Industry 4.0 and CE and identifying future research directions	Investigate the impact of different Industry 4.0 technologies on the CE Lack of empirical investigations on how CE and Industry 4.0 principles are employed in practice by firms Understanding the role of Industry 4.0 to support stakeholders involved in circular business models
Centobelli et al. [25]	2020	Circular business models	To explain how businesses design their business model according to the CE principles	A theoretical, conceptual framework considering the relevance of business models’ challenges	The study of managerial commitment, organisational culture, external environment, and digital technologies that enable the effectiveness of CE business model design
Morseletto [115]	2020	CE	To investigate which targets can accelerate the shift toward the CE	Current targets for recovery and recycling do not necessarily foster CE despite being the most often used targets Identification of novel and existing targets and their role in minimising waste, closing production loops, boosting efficiency, and optimising retention of the economic value of products and materials	The trade-offs, complementarities, and synergies necessary for the CE achievement Examination of CE targets for specific industries/products, business processes, and product categories Analysis of CE targets in relation to innovations is necessary to achieve CE strategies
Esmaeilian et al. [40]	2020	Blockchain technology	To offer an overview of blockchain technology and Industry 4.0 for moving supply chains toward sustainability	Blockchain improves sustainability by developing incentive mechanisms and tokenisation to motivate consumer behaviour, improving visibility across the entire product lifecycle, increasing systems efficiency, and fostering sustainability monitoring and reporting performance across supply chain networks	A limited focus on the social and economic aspects of sustainable blockchain technology More discussion is required to clarify the connection of blockchain technology with other complementary IT infrastructures for the transition toward sustainability and the CE
Sharma et al. [150]	2020	E-waste management	To identify the most influencing key enablers of e-waste management in CE	The environmental management system represents the most critical driving factor to impact all other enablers E-waste management can be efficient if it relies on manufacturing eco-friendly products, formulating strict legislation, developing a green image, and supporting manufacturers to adopt CE practices	The potential of emerging technologies to support e-waste disposal and dumping points and reduce the harmful environmental effects
Vanapalli et al. [166]	2021	Plastic waste management in the COVID-19 era	To highlight the effects of COVID-19 on plastic waste generation To shed light on the issues caused by the pandemic on the existing waste management systems	The COVID-19 pandemic has increased the use of plastics for hygienic and safety purposes Innovation in current technologies and products could help realise sustainability Reduction in the use of single-use plastic could influence consumers’ behaviour	The incorporation of novel technologies into existing management systems to support plastic reuse or recycling The role of policies regarding behavioural and psychological attributes of social awareness, incentives, and public–private investments in infrastructure and research to assure sustainable and inclusive plastic waste management
Ibn-Mohammed et al. [68]	2021	CE and COVID-19	To critically review the negative and positive impact of the COVID-19 pandemic and their role in accelerating the transition toward a more resilient and sustainable low-carbon economy To analyse the downsides of exploiting the pandemic-driven benefits to realise sustainable development goals	There is a need to rethink the current global economic growth model in the interest of a more sustainable approach recalibrated on the CE framework Concrete sector-specific suggestions on CE-based solutions as a driver for the global economic growth and development in a post-pandemic world	The potential of digital technologies in assuring the energy-efficient and low carbon future of the CE ecosystem
Schyns and Shaver [148]	2021	Plastics	To review the existing methods and issues for mechanical recycling	Waste management systems have to be involved when designing blends, polymers, and mechanical recycling activities	The limitations of the mechanical recycling systems in the plastics industry The potential of chemical recycling methods for plastics
Nandi et al. [117]	2021	Blockchain technology	To offer insights from the COVID-19 pandemic for building more sustainable, resilient, and transparent supply chains	Blockchain supports localisation, agility, and decentralisation of supply chains Blockchain enables supply chain traceability and responsiveness in the CE	Case studies of blockchain-enabled circular supply chains and their impacts on localisation, agility, and decentralisation in the CE The role of governments to encourage the adoption of blockchain technology in CE models
Bag et al. [11]	2021	Industry 4.0	To explore how significant an impact Industry 4.0 deployment has on 10 R (refuse, rethink, reduce, reuse, repair, refurbish, remanufacture, repurpose, recycle, and recover) advanced manufacturing capabilities and its implications on sustainable development with the moderation of an Industry 4.0 delivery system	The path degree of Industry 4.0 adoption and 10 R manufacturing capabilities are important Industry 4.0 R advanced manufacturing capabilities have a positive effect on sustainable development goals Industry 4.0 delivery system has a moderating impact on the path degree of Industry 4.0 application and 10 R advanced manufacturing capabilities	Human resources management in the context of Industry 4.0 and the CE The role of artificial intelligence in supporting 10 R advanced manufacturing and advancing Industry 4.0 and sustainability
Bag et al. [9]	2021	Big data analytics, AI, and CE	To apply institutional theory and resource-based view theory to explain how automotive companies configure tangible resources and employees’ skills to reinforce technological enablement and enhance sustainable manufacturing activities and develop CE capabilities	The positive relationship between big data analytics (BDA)-artificial intelligence (AI) adoption and sustainable manufacturing practices and CE capabilities	The relationship between blockchain technology-Internet of Things adoption and sustainable manufacturing practices and CE capabilities The relationship between the additive manufacturing implementation and sustainable manufacturing practices and CE practices
Yang et al. [175]	2021	Lithium-ion battery	To explore technologies and research endeavours in battery recycling from the view of economic viability and lifecycle inventory	A commentary on the issues facing battery recycling and the importance of battery design and CE in achieving the sustainable development of the battery industry, in which manufacturers, consumers, and governments are actively involved	Examination of the role of governments, the general public, and the manufacturers in promoting the CE concept in the battery industry Solutions to make feasible, commercially viable, and profitable CE business models in the battery industry
Chen et al. [27]	2021	Plastics	To discuss how single-use plastics are landfilled or incinerated, causing pollution and environmental degradation To review single-use pollution contamination in different environmental media such as rivers, soils, oceans, and lakes around the world	There is a growing trend to reduce single-use plastics Identification of regulatory tools and voluntary actions to minimise utilisation of single-use plastics	Lack of effective solutions to recycle single-use plastics Measures to lessen the impact of single-use plastics pollution during the transition toward the CE
Kumar et al. [90]	2021	Food waste	To assess the techno-economic and environmental influences of biochar synthesis and its emerging application for biogas production	Enhanced stability and reliability of anaerobic digestion with biochar reflects a new paradigm to generate renewable energy, produce fertiliser-grade digestate, and reduce waste The integrated anaerobic digestion and pyrolysis system would lead to further optimal environmental performance and economic feasibility	The implementation of the CE model for the AD digestate management to close the loop of materials flows Identification of best practices of sustainable digestate management with CE models
Rajput and Singh [132]	2021	Industry 4.0	To identify the Industry 4.0 barriers to realising CE	The digitalisation process and semantic interoperability represent critical barriers with high driving power and low dependence Other critical Industry 4.0 barriers to achieving CE also include cyber-physical systems standards and specifications, sensor technology, and design issues	Applications of quantitative and qualitative techniques to analyse the enablers of Industry 4.0 technologies for the CE transition The barriers to Industry 4.0 technologies and solutions for their deployment in CE applications

## Findings of MPA

**Fig. 4:**
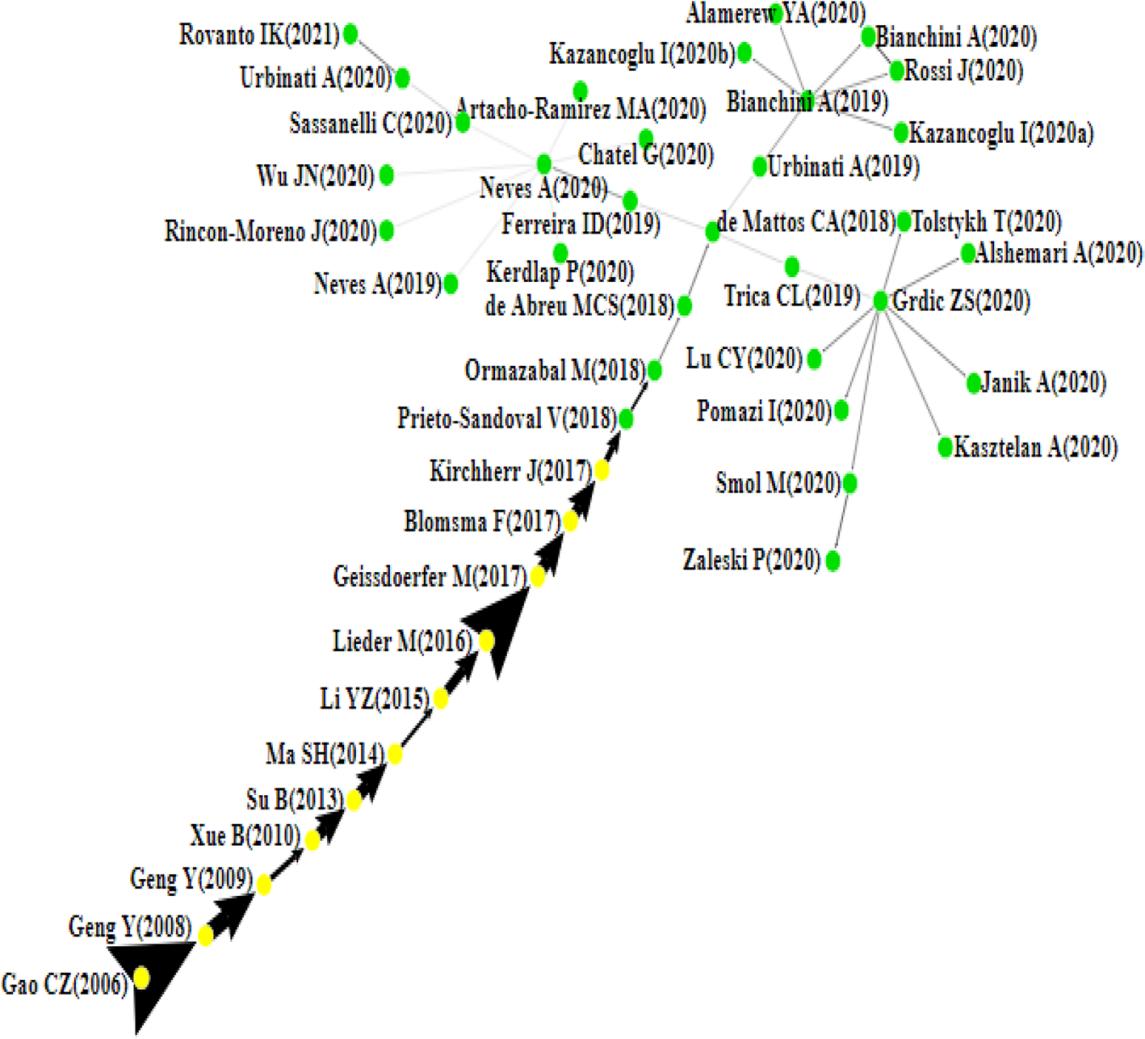
Forward local main path

**Fig. 5:**
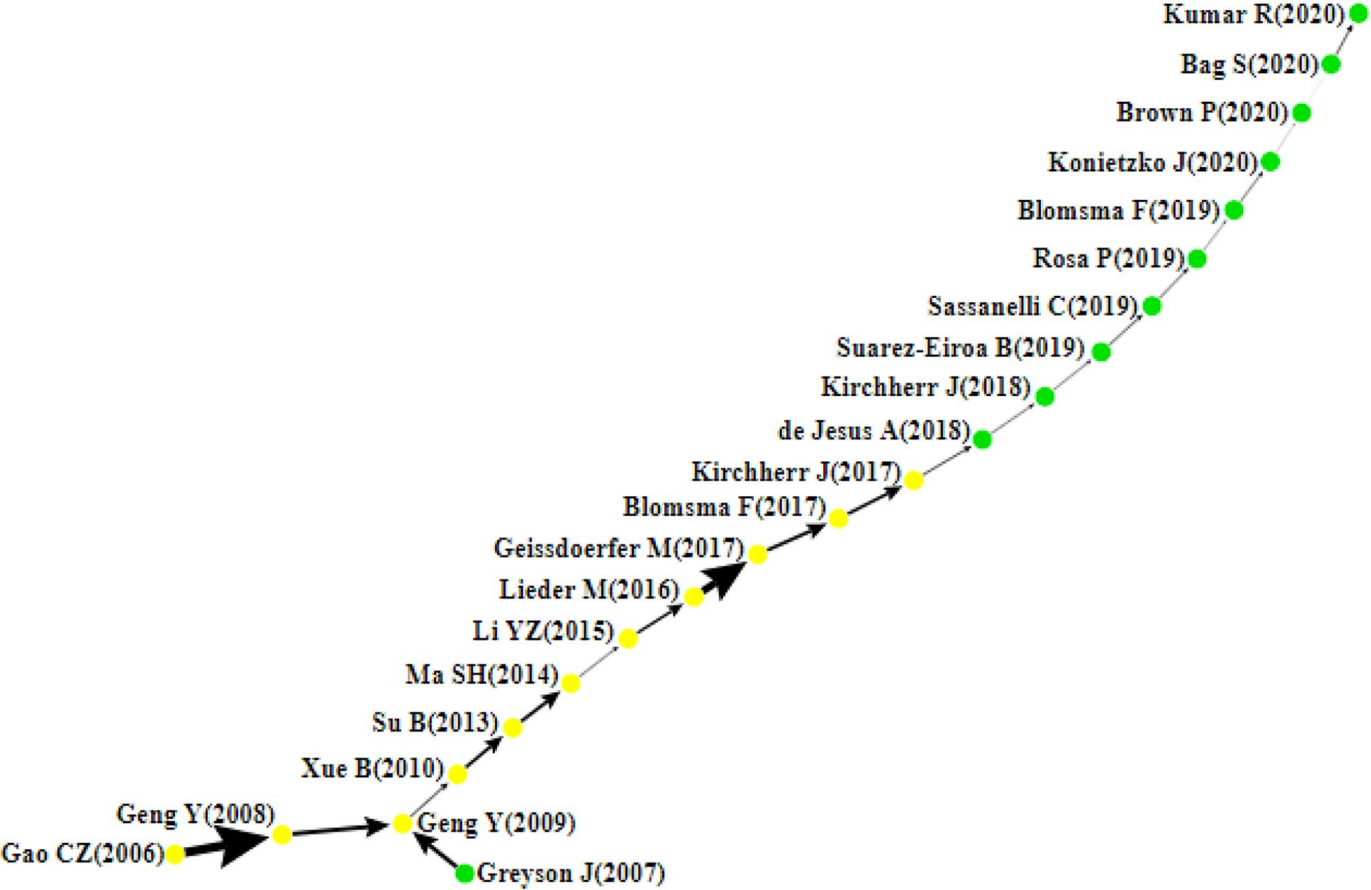
Backward local main path

**Fig. 6:**
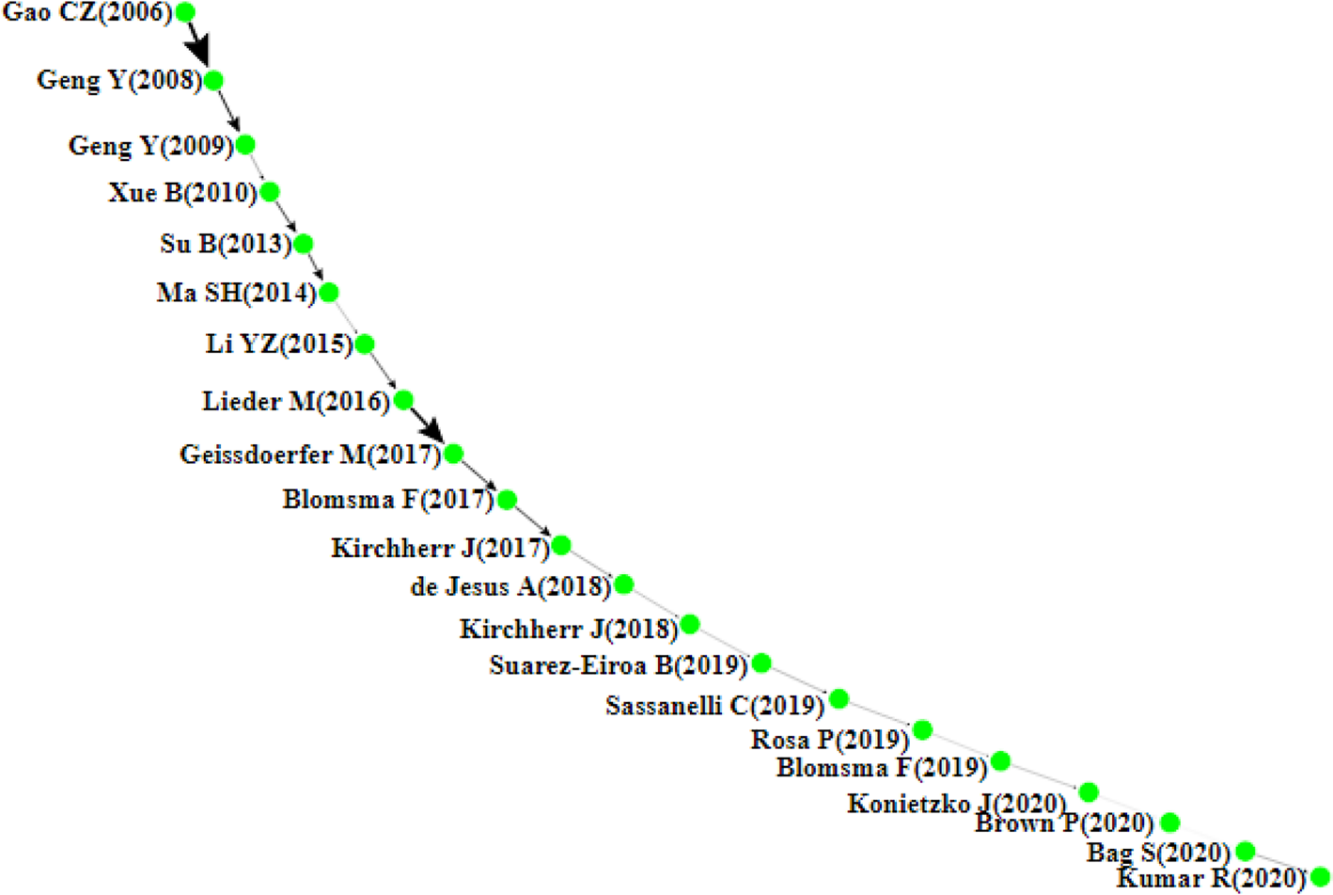
Global main path

**Fig. 7:**
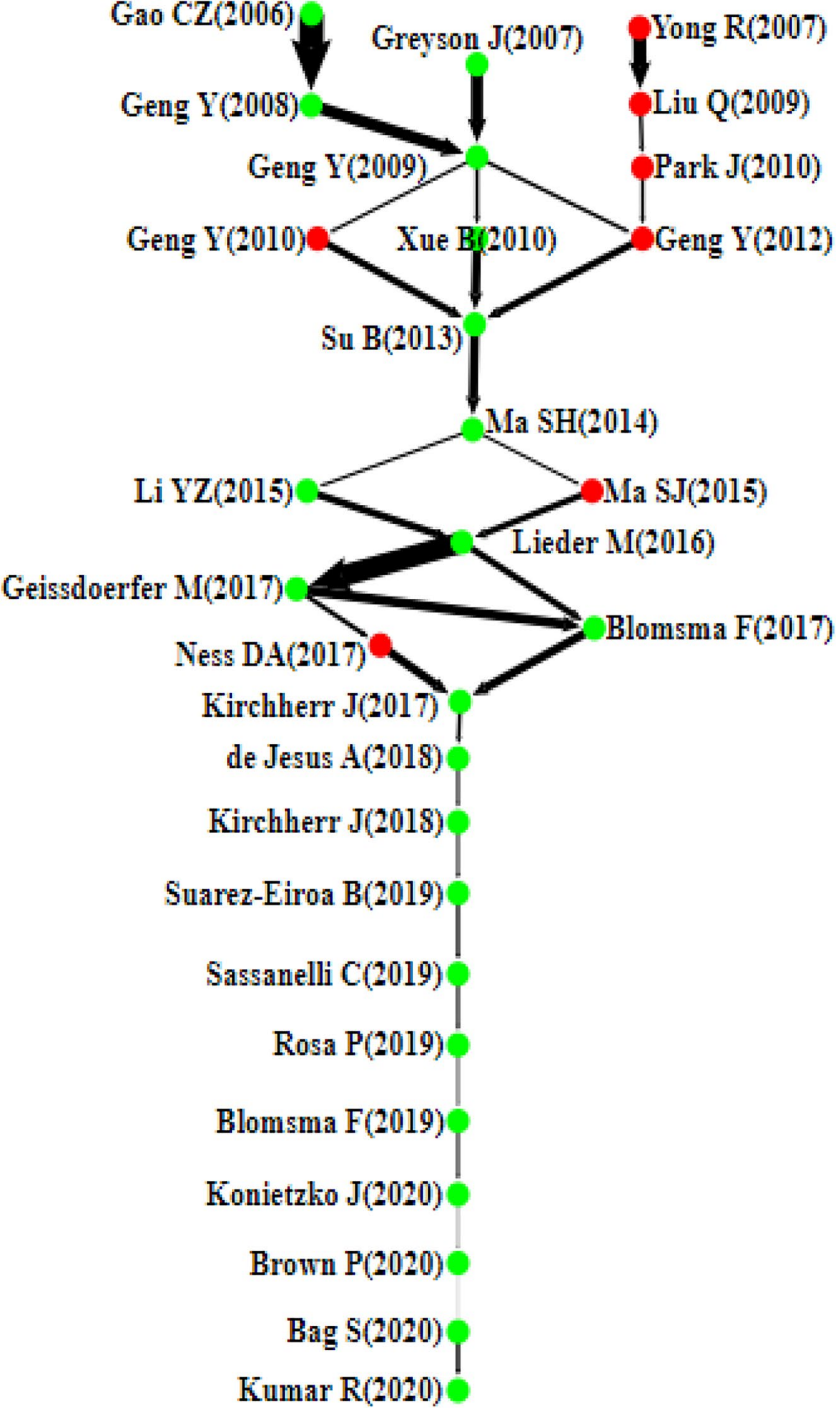
The key-route main path

This section presents the citation network analysis of the chosen articles and then discusses the findings of the four main paths, namely the local (forward, backward), global, and key-route main paths (see Figs. 4, 5, 6, 7). Each node in these graphs represents a single article and is accompanied by the author’s information and the publication year. The direction of knowledge flow is represented by the arc, and the thicker the arc, the greater its significance.

### The Citation Networks

**Fig. 8:**
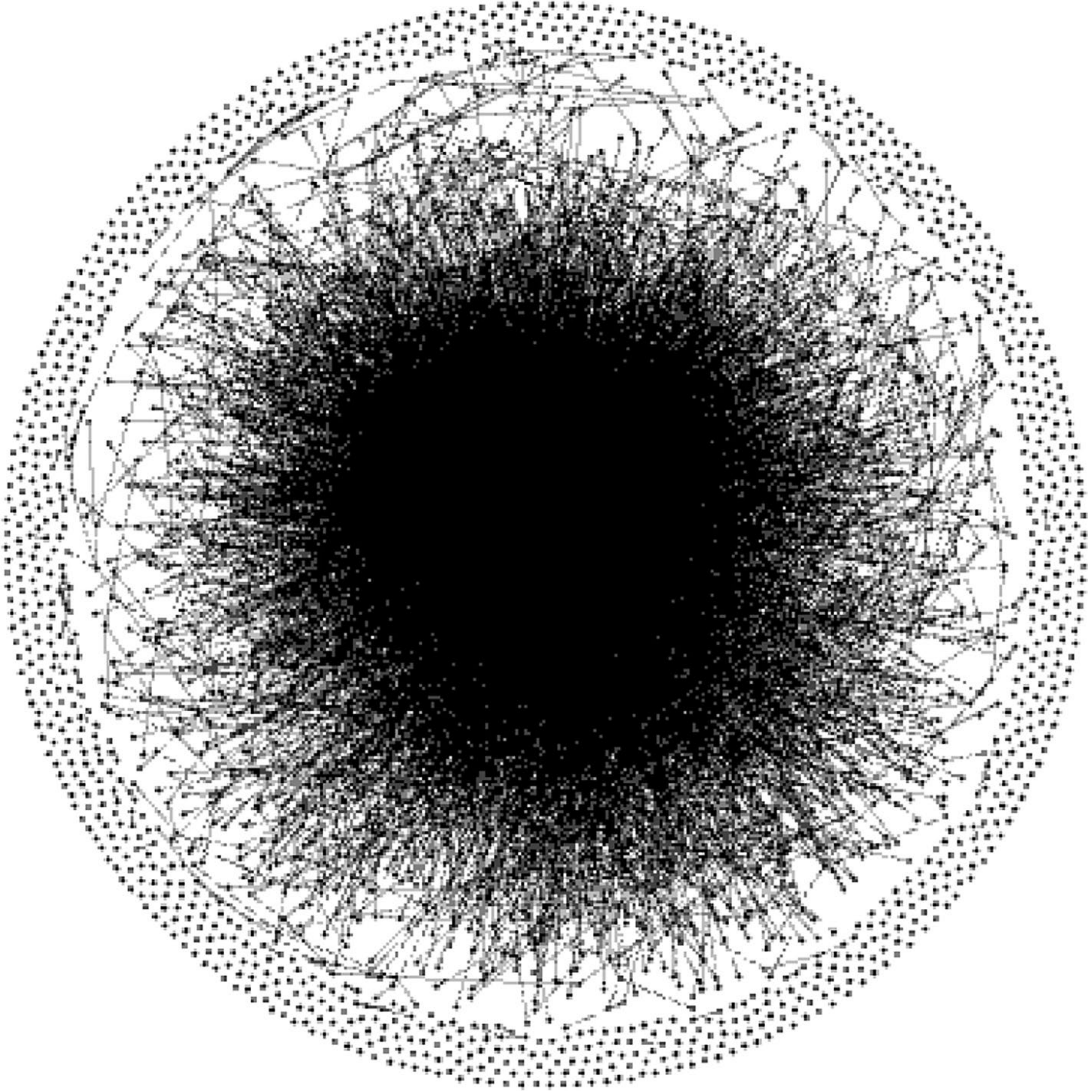
The citation network of selected articles

Figure 8 depicts the entire citation network for the selected articles. There are 5431 nodes and 26,448 links that make up the network. There are three types of nodes. The first category consists of nodes in the largest subnetwork. The subnetwork consists of 4363 nodes that are closely interconnected, thereby constituting the core of the entire network. In the following sections, the main paths are derived from the subnetwork that maintains the greatest number of citation relationships between articles. The second category of nodes consists of those with few citations. These nodes are dispersed on the network's periphery and are connected to multiple articles. The third type consists of nodes that are disconnected from all other nodes in the network.

### The Local Main Path

Figures 4 and 5 present the local main paths, comprising 43 and 22 articles, respectively. In total, 11 common articles appear on both paths; meanwhile, nodes coloured in green are different. It is evident that Gao et al. [47] represent the first article on both paths, which is of vital significance in the initial development of the CE field. In this study, the authors develop a three-level education framework to meet the theoretical and technological requirements for implementing the CE and achieving a sustainable society. Geng and Doberstein [53] further examine the transition toward the CE. They discuss the measures being deployed in China for the long-term promotion of CE, including the development of objectives, legislation, and policies and measures. Similarly, Geng et al. [56] describe the approach used in achieving the CE concept in China and the barriers that have held back the complete implementation of CE, including lack of incentives for older industries, lack of financial support, and low public awareness and participation in CE initiatives. In Xue et al. [172], the authors investigate the barriers to implementing the CE in China and find that there is limited awareness of CE among government officials and reluctance to pay more money for environmentally friendly products. The backward local main path reveals another article at the start of the path, Greyson [63], which introduces an approach designed to prevent waste and other global impacts based on established recycling practices, CE policy, and recycling insurance. In Su et al. [155], a holistic review of the CE is conducted to clarify how this strategy has been developed and implemented.

As an innovation-driven approach, the CE is driven by the continuous sustainability transition that impacts virtually all players across many industries. For example, Ma et al. [108] focus on private steel firms in China and offer a holistic perspective on the CE mode inherent in this economic sector, explaining how important reductions in energy consumption and pollutant emissions have been achieved. In the papermaking industry, Li and Ma [96] examine the contribution of the CE to cleaner production, energy saving, emission reduction, and sustainable development. In the manufacturing industry, Lieder and Rashid [98] review the CE landscape in the context of resource scarcity, waste generation, and economic advantages. It is argued that joint support of all actors is imperative to achieve the CE on a large scale successfully. In the following year, three papers, Geissdoerfer et al. [52], Blomsma and Brennan [18], and Kirchherr et al. [84], concentrate on reviewing and conceptualising the CE holistically.

After Kirchherr et al. [84], the two local main paths start to be different. In the forward local main path, Prieto-Sandoval et al. [130] propose a consensus view of the fundamental notions of the CE framework and emphasise its relation with eco-innovation. In Ormazabal et al. [124], the authors carry out an empirical investigation aiming to explore the potential for the adoption of the CE in small and medium enterprises and the challenges and prospects they may encounter. The findings of the study point to three factors associated with the firms’ perception of the CE, including material provision, resources’ reuse, and financial advantage. The following paper on the path is by de Abreu and Ceglia [2], who examine the role of institutional capacity-building through industrial symbiosis in implementing a CE and find that government is a primordial actor in establishing and maintaining an industrial symbiosis coordination network. In De Mattos and De Albuquerque [37], the authors discuss the enabling factors and strategies for developing and spreading circular business models.

After this study, research at the end of the forward local main path becomes more prosperous and splits into three branches. The first branch begins with the article of Trica et al. [161], in which a methodology for studying the sustainability of the CE model is presented based on environmental factors such as resource productivity, environmental employment, recycling rate, and environmental innovation. Later, Sverko Grdic et al. [158] explore the implementation of the CE in member countries of the European Union and conclude that the transition toward this approach can ensure economic growth, increase GDP growth, reduce the use of natural resources, and guarantee greater environmental protection. After [158], several studies have emerged recently.

For example, three papers, Smol et al. [154], Zaleski and Chawla [181], and Pomázi and Szabó [129], examine the transformation process toward the CE in Poland and other Visegrad countries, namely, the Czech Republic, Hungary, and Slovakia. On a broader scale, Janik et al. [70] and Kasztelan [74] argue that the achievement of climate neutrality and social sustainability in the EU necessitates explicit measures to limit greenhouse gases (GHG emissions) and implement CE principles more practically. In response to resource scarcity and environmental degradation issues, the CE represents a comprehensive solution to achieve closed-loop material flows in the entire economic system and foster industrial sustainability. In this regard, Tolstykh et al. [160] note that industrial ecosystems established based on a symbiotic model and applying CE principles can maximise resource use efficiency, reduce industrial waste, and achieve holistic sustainability. Likewise, [104, 105] assert that the development of an industrial CE encourages the efficient recycling of resources, supports industrial transformation and upgrading, and accelerates the realisation of the CE. Addressing the sustainability issues in the pharmaceutical industry, Alshemari et al. [7] study whether the adoption of CE principles can reduce pharmaceutical waste, maximise medicines’ value, and foster sustainability. After reviewing the literature, the authors conclude that CE principles can improve pharmaceutical waste management and cost and minimise the harmful environmental impacts of unsafe disposal.

The second branch starts with the paper of Urbinati et al. [164], who look into the managerial practices that firms embrace in their business model to introduce circular products. According to their study, the adoption of CE at the product level, energy efficiency and use of renewable energies, product and process optimisation for resource efficiency, product design for circularity, and transformation of waste into a resource facilitates the transition toward the CE and the introduction of circular products. In Bianchini et al. [17], a new circular business model visualisation tool is proposed for potential circular opportunities and to determine the best CE strategy. Five papers are detected on the main path including Bianchini and Rossi [16]. They introduce an integrated, industry-oriented methodology to aid knowledge transfer from academia to industry and support businesses in assessing technical, economic, environmental, and social benefits or risks of phosphorous recovery. Kazancoglu et al. [78, 78], in their two papers, conceptualise and investigate the barriers to circular supply chains in the textile industry using a stakeholders’ perspective. The two sink papers closing this branch guide organisations to adopt circularity strategies at the product level [4] and leverage intelligent assets from Industry 4.0 to improve circular business models [141].

The third branch starts with Ferreira et al. [43], which studies the CE in Portugal and Spain’s pulp and paper sectors. The following paper, Neves et al. [120], reviews the trend of industrial symbiosis research and maps the current case studies worldwide. This study is disseminated into several papers highlighting the importance of industrial symbiosis as a strategic tool for increasing resource productivity and creating new business opportunities. For example, Neves et al. [121]^1^ state that industrial symbiosis can bring several environmental, economic, and social benefits, yet this potential is determined by several drivers such as the diversity of industries, geographic proximity, supporting entities and legislation, measures, and policies. In Kerdlap et al. [81], the authors review industrial symbiosis network (ISN) facilitation tools, including lifecycle assessment and lifecycle costing. Moving further in this direction, Wu and Jin [170] examine the influence of symbiotic measures on the evolution of structure and function of the iron and steel industrial symbiosis network, concluding the importance of these measures to ensure low carbon design and management. Two papers, Rincón-Moreno et al. [138] and Artacho-Ramírez et al. [8], conduct case studies to analyse the potentials and challenges of industrial symbiosis in Spain. Finally, a bifurcation is initiated by Sassanelli et al. [146] research. They synthesise the literature surrounding the Design for X (DfX) approaches necessary for the development of circular solutions. Building on this paper, Urbinati et al. [165] and Rovanto and Bask [142] investigate the adoption of the CE at a company, supply chain, and broader level (i.e. society).

On the backward local main path, ten different papers appear. de Jesus et al. [71] review eco-innovation and the CE and deduce that the transition toward the CE depends on systemic eco-innovation. Kirchherr et al. [83] examine the CE barriers in the EU and find that cultural barriers, especially the lack of consumer interest and awareness and organisational culture, are the most significant. The following three papers, Suárez-Eiroa et al. [157], Sassanelli et al. [145], and Rosa et al. [139], are reviews of operational principles of CE, CE performance assessment methods, and circular business models and their classification methods. Furthermore, the realisation of the CE relies on the drive to innovate and create value propositions by offering new products and services which preserve the natural environment and improve social welfare. Considering innovation as a key element for the success of the CE, Blomsma et al. [19] posit that the support of innovation processes can help translate the CE concept into practice. Konietzko et al. [85] insist on incorporating ecosystem perspectives into circular-oriented innovation and developing more practitioner-focused tools for circular ecosystem innovation. In Brown et al. [24], collaborative circular-oriented innovation practices are investigated, and the findings illustrate the value of open innovation to enable the CE. At the end of the backward local main path, the last two papers study the relationships between Industry 4.0 and the CE [10, 92].

Summarising, the local main paths show that most articles focus on conceptualising the CE and identifying its drivers and barriers, while few articles concentrate on the contribution of Industry 4.0 technologies to the acceleration of CE transition [9, 11, 34, 92, 118, 131, 162, 180]. Therefore, future studies should assess how Industry 4.0 technologies effectively unlock the CE implementation, support the development of circular business models, and monitor product lifecycle [118, 140, 173]. Given that the progress of industrialisation can result in the exponential boom of several markets, including electronics, it is still unclear how product obsolescence [29, 66] due to the acceleration rate of technological developments or the artificial limitation of product durability can hamper efforts to operationalise the CE and lead to unstainable production and consumption models.

### The Global Main Path

The previous main paths identify local influential arcs in the citation network. In contrast, the global main path can find the largest and most influential path, as shown in Fig. 6, containing 21 significant articles. The SPC values of all arcs on this path are presented in Table 3. The arc from Gao et al. [47] to Geng and Doberstein [53] has the largest SPC value, followed by the arc from Lieder and Rashid [98] to Geissdoerfer et al. [52] and the arc from Geng and Doberstein [53] to Geng et al. [56]. From the global main path, it is possible to identify the seminal articles contributing to the evolution of knowledge in the CE domain.

**Table 3:** The SPC values of all links of the global main path

Links	SPC	Ranking
Gao CZ (2006)—> Geng Y (2008)	200,162,914	1
Geng Y (2008)- > Geng Y (2009)	94,937,918	3
Geng Y (2009)- > Xue B (2010)	30,552,570	10
Xue B (2010)- > Su B (2013)	70,585,660	6
Su B (2013)- > Ma SH (2014)	74,016,287	5
Ma SH (2014)- > Li YZ (2015)	22,100,799	14
Li YZ (2015)- > Lieder M (2016)	58,569,516	8
Lieder M (2016)- > Geissdoerfer M (2017)	166,232,292	2
Geissdoerfer M (2017)- > Blomsma F (2017)	75,862,270	4
Blomsma F (2017)- > Kirchherr J(2017)	68,829,780	7
Kirchherr J (2017)—> de Jesus A(2018)	30,855,768	9
de Jesus A (2018)—> Kirchherr J(2018)	21,751,793	15
Kirchherr J (2018)—> Suarez-Eiroa B(2019)	19,535,230	17
Suarez-Eiroa B (2019)—> Sassanelli C (2019)	26,170,403	12
Sassanelli C (2019)- > Rosa P (2019)	22,945,155	13
Rosa P (2019)- > Blomsma F (2019)	14,324,205	18
Blomsma F (2019)- > Konietzko J (2020)	20,863,215	16
Konietzko J (2020)- > Brown P (2020)	7,247,493	19
Brown P (2020)- > Bag S (2020)	2,993,803	20
Bag S (2020)- > Kumar R (2020)	28,924,431	11

The visual inspection of the citation path reveals that the thickness of links is more noticeable at the beginning of the path than at the end of the path, implying that earlier articles receive significant attention. However, articles at the end of the path still have a relatively low number of citations. While they are recent, the appearance of these articles at the end of the path reflects their importance as main followers of mainstream works in the citation network. Therefore, the position of these articles still needs to be evaluated with the passage of time and the publication of more articles on the topic. Overall, the global main path aims to offer a different angle to the overview of the CE domain while not meant to be comprehensive [179]. Indeed, several important papers are missing from this path, and this shortcoming can be addressed by the previous local main paths and key-route main path.

### The Key-Route Main Path

The key-route main path enables a more detailed extraction of the information about the evolutionary structure of the CE domain. In addition, this main path can reveal the convergence and divergence phenomenon of the CE scientific development. This study uses the local method, chooses the number of key-routes with a step size of 5, and eventually selects 15 for the best result. Figure 7 shows the key-route main path, which portrays the repeating cycles of converging and diverging. Articles on this path are almost similar to the ones appearing on the local main paths, except for seven new papers (represented by red nodes). This section presents the analysis of these articles and the divergence-convergence patterns of CE development.

At the beginning of the path, papers on the right side have not appeared on the above three paths. Yong [176] explains the CE concept, the reasons behind China’s full effort to promote the CE, and how to make it realisable in the country. Inspired by the research from these authors, Liu et al. [101] and Park et al. [128] attempt to understand the level of public awareness and the performance of a CE and the challenges and opportunities of achieving economic growth environmental stewardship in China’s CE.

The first divergence-convergence pattern is at the start of the key-route main path. Papers diverge at Geng et al. [56] and finally merge at Su et al. [155]. Building on Geng et al. [56], Geng et al. [55] suggest a new method based on emergy analysis and synthesis, taking into account the contribution of ecological products and services during the evaluation of industrial parks eco-efficiency. Later, Geng et al. [54] introduce a national CE indicator system to evaluate the holistic performance of China’s CE efforts. Overall, several findings can be obtained from this part. For instance, at the early stage of the development of the CE domain, studies focus on conceptualising the CE and investigating the opportunities and barriers to its implementation in China. Despite achieving great advances in renewables utilisation, the high level of industrialisation in China is controversial due to its significant negative impact on the efficiency of the industrial CE [104, 105]. Moreover, papers lack a comparative approach and cross-cultural analysis to derive lessons from CE implementation in different geographical contexts [50, 54, 114, 172]. Studies on the situational and organisational factors that address the barriers to the realisation of CE plans are also needed [3, 69, 110].

The second divergence-convergence pattern diverges at Ma et al. [108] and gathers at Lieder and Rashid [98]. Several important features exist in these four scholarly contributions. First, the dominant method used is case studies, and the geographic context is China. The case study research provides a detailed understanding of the complicated concept of CE using a limited number of observations and building on the articles of the predecessors. Second, these papers focus on the iron and steel, papermaking, chemical, and manufacturing industries. For example, Ma et al. [107] provide a combination of tools to aid decision-makers in addressing resource and ecological efficiency issues, thereby supporting the sustainable development of resource-based chemical industries. Third, there is a lack of studies on the role of some industries, particularly those featured by low environmental pressure and high added value, in supporting the realisation of the CE [177]. The circularity of materials, resources, and waste in the light of the increasingly global and complex industrial activities is a challenging task [12]. While one of the promises of the CE is to reduce emissions, landfills, and waste, there is a potential for integrating new technologies such as the internet of things, artificial intelligence, blockchain, and big data analytics to facilitate reuse, remanufacturing, and recycling operations [76, 134, 136].

The last divergence-convergence pattern diverges at Lieder and Rashid [98] and eventually merges at Kirchherr et al. [84]. After analysing this part, the following findings can be observed. First, articles on this part mainly revolve around reviewing the CE literature and establishing a set of terms, premises, and definitions for the CE. For example, some of the influential reviews in the CE domain include Lieder and Rashid [98] and Geissdoerfer et al. [52], from which another review by Ness and Xing [119] emanates. In this latter, the authors synthesise the literature on the nexus of CE and industrial ecology and their application in the building sector, thereby promoting resource efficiency and the sustainable and carbon–neutral building agenda. It can be seen that reviews on the CE are abundant, yet an approach to studying the knowledge diffusion in this field is still missing, making the present study the first to use MPA in the CE literature. The convergence to the study of Kirchherr et al. [84] indicates that most previous papers on the path are reviewed therein.

## Discussion

Increasing emphasis on the CE domain necessitates a comprehensive examination of the entire field. Using a content analysis based on keyword co-occurrence clustering and the most recent articles published in 2020 and 2021, this paper attempts to analyse the most important research topics in the CE field. Through MPA, additional useful information can be uncovered. This study analyses research topics within the citation network of 5431 articles that discuss the CE paradigm. Four types of main paths, including local (forward, backward), global, and key-route main paths, were established to reveal the knowledge diffusion trajectories of the CE domain from various vantage points. The analysis of the local main path is used to visualise and present the historical reconstruction of the CE region. In addition, the global main path is utilised to determine the most influential path in general, followed by the key-route main path, which reveals the evolutionary structure of the CE field in its entirety. According to the insights provided by the clustering of keyword co-occurrences, the content analysis, and the MPA conducted, the following are the key findings and implications:

### Main Findings

- The keyword co-occurrence network analysis indicates that CE research has focused on several topical themes, including CE and sustainability, bioeconomy, CE practices, LCA and industrial symbiosis, construction activities, and waste management. Research about the relationships between CE and sustainability occupies a high percentage. As a paradigm shift, this study argues that the CE can positively contribute to sustainable development goals, close the resource loops, and achieve a harmonious integration of environmental protection, economic performance, and social sustainability to present and future generations [52]. Related keywords to CE and sustainability include sustainable development, SDGs, Industry 4.0, circular business models, environment, and remanufacturing.
- The content analysis of the most recent influential papers complements the findings of the keyword co-occurrence clustering. It reveals an ongoing focus on specific topics, including biorefineries, plastics, food waste, and e-waste management. In addition, researchers have concentrated on the potential of Industry 4.0 technologies to accelerate CE implementation. These include blockchain technology, big data analytics, and artificial intelligence. The recent COVID-19 outbreak also underlines the need to develop more sustainable production and consumption systems to recover from the adverse effects of the pandemic and bring normalcy to business activities and the economy.
- The citation network of selected articles is clustered according to three types of nodes: The first one indicates the largest subnet, and all nodes are closely linked. Therefore, such nodes represent the core of the entire network. The second type has several citations scattered around the network. Finally, the third type represents isolated nodes disconnected from any node in the network. Combining three types of main paths makes it possible for us to systematically grasp the evolution of the CE research in the past five decades (1970–2020), uncovering the points that have made major contributions to the development of the CE domain.
- The local main path consisted of 43 and 22 articles, with eleven common articles appearing in both paths (forward and backward). The development of the CE field was initiated with one paper in 2006, which appears in both parts. The backward local main path mainly focused on the theoretical and technological requirements of the CE implementations. In contrast, the forward local main path from 2018 focused on the fundamental notions of the CE frameworks and eco-innovation. For instance, de Jesus et al. [71] reviewed eco-innovation and CE. Three branches along the same path emerged in the forward local main path after De Mattos and De Albuquerque [37]. The first branch analysed the sustainability issues of the CE principles based on environmental factors. The second branch concentrated on managerial practices within circular business models. The third branch focused on industrial symbiosis research using lifecycle assessment tools.
- On the other hand, the global main path was used to find the most influential path, which contains 21 significant articles. The visual inspection of the citation path revealed that the first articles received more attention than the newly published ones, based on the thickness of the links. However, newly published articles still have low citation rates at the end of the path. Therefore, these articles need to be evaluated over time to understand their position on the path. From the structure of the main paths, the global main path highlights three influential papers, including Geissdoerfer et al. [52], Blomsma and Brennan [18], and Kirchherr et al. [84], which systematically discuss the state-of-the-art of CE research in the early stage.
- More details are also revealed with the analysis of the key-route main path. The key-route main path was used to present more detailed information regarding the evolutionary structure of the CE domain by describing the repetitive cycles of divergence and convergence. The analysis of the key-route main path showed three divergence-convergence patterns. The first divergence-convergence pattern is at the start of the key-route main path. This part starts with the study of Geng et al. [56], which plays an important role in integrating different concepts and inspiring new ideas in knowledge transmission, which is the core work in the domain of CE. Overall, articles in the first cycle of the key-route main path focus on conceptualising the CE and explaining the drivers and barriers of its implementation in some geographic contexts. The second divergence-convergence contains studies that discuss the CE in some industrial contexts such as the iron and steel, papermaking, chemical, and manufacturing industries. However, the third divergence-convergence part comprises several review papers serving as references in the CE field. In general, the key-route main path accentuates the need for research on selecting appropriate culture for CE and the impact of government intervention on the CE implementation [143]. In this context, the decision-making process needs to be studied further by considering the influence of government [22].

### Future Research Directions

In recent years, the CE domain has received increased focus; studying the CE domain systematically and exhaustively provides useful information for future research. Using the MPA method and 5431 articles published between 1970 and 2020 that were retrieved from WoS, this paper attempts to provide deeper insights into CE research from a dynamic perspective. Through the combination of local, global, and key-route main paths, this paper reveals the knowledge diffusion trajectories within the CE domain. This study suggests future CE research directions based on its primary findings:

- There is a need to examine the CE beyond the organisational level, considering the entire ecosystem [68]. This is crucial as the CE constitutes a holistic philosophy that aims to form industrial symbiosis networks and relationships involving several organisations and stakeholders [77]. Therefore, the CE evaluation and assessment methods should be designed to meet the requirements of the different stakeholders, including designers, regulatory bodies, policymakers, and customers [26].
- Future research can adopt case studies and conduct more evidence-based research [122, 126]. Existing studies provide CE conceptualisations, frameworks, systems, and archetypes [51]. Considering the activities of companies, more empirical studies are necessary to investigate collaborative relationships and innovation in CE implementations [20].
- Recently, CE has attracted growing popularity worldwide because of its alignment with sustainability and the role of CE-based business models in improving economic growth, sustainable development, resource use, and the environment [10]. For example, Geissdoerfer et al. [52] argue that sustainability and the CE concept are fundamentally global and commonly share several concerns related to the current state of technological deployments, industrial production, and consumption, which might threaten future generations and unearth sources of unexploited competitive advantage. More attention needs to be paid to the intersection between sustainability and the CE [52, 88, 147].
- With the emergence of Industry 4.0, new technologies are causing significant disruptions and forcing the CE field to develop new business strategy models [118, 140, 180]. The MPA reveals that the CE literature has been recently focused on Industry 4.0 technologies and their contributions to the CE [51]. Therefore, future research directions in the CE domain may focus on more technology-related topics [89]. Furthermore, the integration of emerging concepts and techniques such as big data, the internet of things, artificial intelligence, and blockchain is expected to provide new prospects and challenges for the future development of smart circular supply chains [75], 134, 136 considering specific CE principles [60]. Notably, the smart and sustainable circular economy model, which includes the concepts of smartness, sustainability, and circularity [75], will gain more attention in future research. This model will be adapted and tested for different industry areas within the CE domain.

### Potential Subject Areas

Research on the CE has received significant attention from different fields and disciplines. In order to determine which subject areas, 5431 articles were analysed. Table 4 shows the subject areas according to the number of articles. Articles in the field of environmental sciences; green and sustainable science and technology; engineering, environmental; and environmental studies appear to have a high percentage, representing a significant portion of the total articles selected. Interest in these four subject areas has grown over the years, and academics are expected to continue to study the CE from environment, sustainability, and technology perspectives. There are also publications from other subject areas such as energy and fuels (3.38%); engineering, chemical (2.56%); chemistry, multidisciplinary (2.45%); materials science, multidisciplinary (2.27%); and management (2.12%). Although CE research penetrates environment, sustainability and technology subject areas, contributions from engineering, manufacturing, and food science and technology subject areas are needed.

**Table 4:** Classification of articles according to subject areas

Subject	Total number of publications	%
Environmental sciences	2672	23.23%
Green and sustainable science and technology	1745	15.17%
Engineering, environmental	1547	13.45%
Environmental studies	768	6.68%
Energy and fuels	389	3.38%
Engineering, chemical	295	2.56%
Chemistry, multidisciplinary	282	2.45%
Materials science, multidisciplinary	261	2.27%
Management	244	2.12%
Biotechnology and applied microbiology	178	1.55%
Business	173	1.50%
Economics	135	1.17%
Metallurgy and metallurgical engineering	118	1.03%
Engineering, manufacturing	117	1.02%
Physics, applied	117	1.02%
Chemistry, physical	114	0.99%
Engineering, industrial	110	0.96%
Water resources	107	0.93%
Food science and technology	104	0.90%
Operations research and management science	102	0.89%

## Conclusion

To achieve sustainability, the CE has attracted widespread interest from both researchers and practitioners. This study employs keyword co-occurrence network analysis and MPA on a sample of 5431 articles selected from WoS between 1970 and 2020. The first approach identifies six research themes in the CE domain: CE and sustainability, bioeconomy, CE practices, LCA and industrial symbiosis, construction activities, and waste management. The use of MPA enables us to identify the seminal papers that have made significant contributions and shaped the CE field, as well as the trajectory and structure of knowledge dissemination over the past half-century. In addition, four distinct main paths are analysed: the local (forward and backward), global, and key-route main paths.

The analysis of the four major paths confirms that the drivers and barriers of CE have been a prevalent topic in recent decades. Publications on the forward local main path tend to concentrate on requirements for implementing CE, such as policy formulation, financial support, and awareness of CE. Additionally, research focused on the enabling factors and promotional strategies for the CE. Eco-innovation, financial resources, government support, and industrial symbiosis are examples. Recent publications on the forward local main path have begun to emphasise the development of sustainable CE models, the practical implementation of the CE in a variety of geographical contexts, and the advantages of industrial ecosystems. The path reveals the coverage of various topics addressing circular business models and supply chains, the implementation of Industry 4.0 in circular strategies, and the vitality of industrial symbiosis. In contrast to the forward local main path, the backward local main path contains studies examining the benefits of CE implementation in various industries, such as the steel and papermaking industries. The relationships between eco-innovation and the CE, the barriers to implementing the CE, and circular business models and innovations were also the subject of additional research along this path. By conducting the global main path, this review identifies that Gao et al. [47] and Geng and Doberstein [53], as the two first articles on the beginning of the path, discussed the requirements, measures, and objectives for CE implementation. Besides the local main path analysis, the key-route main analysis is performed to gain more information about the CE domain, revealing new studies on motives to implement CE, public awareness of this model, and the hurdles facing the CE transition in China. By analysing the knowledge divergence-convergence cycles, CE research has mainly focused on CE conceptualisation in the early stages, then applied case studies in diverse industrial contexts, and finally examined the intersection of CE and industrial ecology in construction. Studies at the end of the path started to focus on the potential of Industry 4.0 for CE.

According to our knowledge, no study has examined the development of CE research utilising the MPA method. Adopting MPA permits the objective and practical identification of some of the most significant contributions to the history and evolution of a domain such as the CE. In addition, by constructing and analysing four different main paths, we hope to represent the prior knowledge flows in the CE domain, thereby educating readers on the evolution of topics and identifying possible future research directions. Consequently, this article provides a comprehensive overview of the CE paradigm and investigates the evolution of the domain, which can be applied to other research domains.

While this article attempts a systematic analysis of the CE domain, certain limitations remain. The fact that we selected and analysed journal articles from a single academic database, Web of Science (WoS), is one of the most significant limitations. Even though journal articles are regarded as a source of certified and high-quality information [133], there are other valuable sources of information. Future research may include other types of publications, such as books, chapters, conference papers, technical reports, and alternative scientific databases, in order to evaluate and improve our research findings by uncovering additional insights, research trends, and theoretical perspectives. A further limitation of the review is that we set a minimum number of keyword occurrences at five. If the value of this threshold is less than or greater than five, the outcomes may vary. Applying other bibliometric techniques, such as bibliographic coupling and co-citation network analysis, to obtain novel insights and research themes can replicate the clustering of relevant literature.

In addition, the authors of this paper selected only CE-related papers from WoS, resulting in insufficient data and the exclusion of other CE-related papers indexed in other academic databases such as Scopus. As a result, the findings may not reveal the complete evolution of the CE domain’s entire spectrum. While the analysis of the four different paths assisted in revealing the knowledge diffusion paths from a variety of perspectives, it is possible that some important publications are not analysed in this review. The MPA examines a few limited works that cannot provide sufficient information on the evolution of the CE field as a whole.

Consequently, future research can consider how to retrieve relevant data and employ an appropriate method to analyse the comprehensive evolution of this scientific field. In addition, the authors did not conduct a bibliometric analysis, which would have been useful for gaining a macro-level understanding of the CE domain’s fundamental information. For example, a bibliometric analysis would have uncovered specific references such as authors, institutions, countries/territories, and journals driving the development of the field and identified potential collaborators for more in-depth research. The combination of bibliometrics and MPA may yield systematic and exhaustive insights into the CE field. Although the four major paths illuminate knowledge diffusion trajectories in the CE domain from a variety of perspectives, this research paper may omit some significant papers. In the future, clustering systems can be combined with the MPA to investigate various trajectories of knowledge diffusion in the CE domain.

## Data Availability

Not applicable.

## Footnotes

1 Neves et al. [121] cites Neves et al. [120] when it was in press.
